# Insulin Blood Levels in Gestational Diabetes Mellitus in Relation to Ethnicity and Age in the Kingdom of Bahrain: A Case-Control Study

**DOI:** 10.7759/cureus.64886

**Published:** 2024-07-19

**Authors:** Tarik AlShaibani, Wadeea Gherbal, Amer Almarabheh, Diaa Rizk, Elaf Alhakmani, Raghad Alshamrani, Farah AlBahraini, Husain Taha, Amal Hassani, Yahya Naguib

**Affiliations:** 1 Department of Physiology, Arabian Gulf University, Manama, BHR; 2 Department of Obstetrics and Gynecology, Salmaniya Medical Complex, Manama, BHR; 3 Department of Family and Community Medicine, Arabian Gulf University, Manama, BHR; 4 Department of Obstetrics and Gynecology, Arabian Gulf University, Manama, BHR; 5 Department of Internal Medicine, Salmaniya Medical Complex, Manama, BHR; 6 Department of Clinical Physiology, Faculty of Medicine, Menoufia University, Shibin El Kom, EGY

**Keywords:** blood glucose, gestational diabetes mellitus, insulin, placental hormones., pregnancy

## Abstract

Background: Gestational diabetes mellitus (GDM) is one of the most common complications of pregnancy. It may be attributed to certain placental hormones during pregnancy which render insulin less effective. Our study aimed to focus on the levels of insulin in gestational diabetic women in the Kingdom of Bahrain as compared with non-diabetic pregnant women. Furthermore, we studied the correlation between insulin levels by ethnicity and age of the pregnant women.

Methods: A case-control study was conducted on 75 pregnant participants: 41 with GDM (test group) and 34 without GDM (control group). Insulin levels were determined in patients with GDM and compared to non-diabetic pregnant women. A comparison between Bahraini and non-Bahraini women was carried out in two different age groups: below and above 30 years of age. P values < 0.05 were considered significant.

Results: The results showed higher mean values of fasting blood glucose (FBG), random blood glucose (RBG), and insulin levels in the test group when compared to the control group. There was no significant difference in FBG, RBG, and insulin levels among Bahraini women with GDM and non-Bahraini women (Indian, Pakistani. Bengali, and Filipino) with GDM. Age, less than 30 vs more than 30 years, had no significant effect on women with GDM.

Conclusion: Insulin levels were higher in pregnant women with GDM irrespective of their ethnicity or age. The lack of blood glucose control in GDM even in the presence of high insulin secretion may suggest loss of insulin effectiveness due to other factors such as stress and lactogenic placental hormones.

## Introduction

Gestational diabetes mellitus (GDM) is defined as a the condition in which insulin cannot be utilized efficiently by the body because of a hormone secreted by the placenta [1]. GDM is currently the most common complication of pregnancy [2,3]. GDM could be explained by the ability of the placental hormonal secretion during pregnancy to deprive the body of a pregnant woman from using insulin efficiently, leading to glucose build-up in the blood instead of being absorbed and then consumed by the cells [4].

GDM is different from type 1 diabetes mellitus where the level of insulin is low due to malfunctioning pancreatic gland. Accumulating data attributed GDM to insulin resistance resulting from placental hormones that are produced during pregnancy. In most cases GDM symptoms disappear following delivery [4]. Insulin is a hormone secreted from the beta cells in the pancreas which constitute 60-70% of the islets of Langerhans. It enhances the cellular uptake of glucose and regulates the blood glucose level [5]. Any abnormality in the production or consumption of insulin can cause diabetes. The International Diabetes Federation estimated that maternal hyperglycemia affected one in every six pregnancies globally [6].

Several factors increase the risk for developing GDM. Having a family history of type 2 diabetes, and a history of GDM in a previous pregnancy are the major risk factors [4]. Other risk factors include prediabetes, impaired glucose tolerance, being overweight, age ≥ 45 years, and lack of physical activity. All these factors may raise the probability of developing GDM [1,2,4]. GDM is associated with several fetal and maternal short- and long-term health risks [7]. GDM is considered an early risk factor for cardiovascular diseases. Over five million women with GDM were associated with an approximately two-fold increase in the risk of developing cardiovascular diseases [8]. Methods of screening include the measurement of fasting plasma glucose (FBG) or random blood glucose (RBG) levels. Women diagnosed by routine blood examination show high glucose levels in the blood, followed by a 75g oral glucose tolerance test (OGTT) to confirm the diagnosis. The recommended screening tests for asymptomatic pregnant women are usually performed between the 24th and the 28th gestational weeks [9,10].

There are many uncertainties regarding the pathophysiology of GDM. One proposed hypothesis suggests the involvement of the placental hormones in maternal metabolic dysfunction and the ineffectiveness of the secreted insulin. Placental secretion of hormones, for instance, human placental growth hormone (PGH) and human placental lactogen (hPL) has been linked to insulin resistance [11]. PGH differs from the pituitary growth hormone in 13 amino acids [12]. PGH increases by six- to eight-fold during pregnancy, and replaces normal pituitary growth hormone in the maternal circulation by the 20th week of gestation [9]. Another hormone that may be implicated in the development of insulin resistance during pregnancy is hPL which is detected in the maternal circulation between the fifth and the seventh gestational weeks. It increases up to 30-fold throughout pregnancy and induces insulin release from the pancreas in pregnancy [13-15]. Lower concentrations of hPL can be also found in the fetal blood [11].

Tumor necrosis factor alpha (TNF-α) is a cytokine and an adipokine that is synthesized and secreted from the placenta and adipose tissue. TNF-α levels have been associated with insulin resistance in obesity, aging, sepsis, and muscle damage. TNF-α inhibits insulin signaling and insulin-regulated glucose uptake, thus suggesting that the insulin resistance of pregnancy may be mediated through this cytokine [16].

In low- and middle-income countries, the standards of antenatal care to detect and manage GDM are often poorly available. Consequently, the prenatal and neonatal burden of GDM may be paradoxically higher in these countries, although this point is not well registered [17]. Our study aimed to detect the levels of FBG, RBG, and insulin in pregnant women with and without GDM in the Kingdom of Bahrain. The effect of age and ethnicity on GDM was also investigated.

## Materials and methods

Ethical considerations

This study received ethical approval from the Research and Ethics Committee at the College of Medicine and Medical Sciences, Arabian Gulf University, Kingdom of Bahrain (E1-P1-10-22). All the procedures were in accordance with the Declaration of Helsinki. Informed consents were obtained from every participant. Every participant was fully informed about all aspects of the study and granted the right to quit. As an incentive, all participants received a copy of their laboratory test results.

Study design

This is a case-control study. The number of participants was calculated in a ratio of two cases: one control according to the availability of cases.

Data collection methods

The data for this study were collected from the Obstetrics and Gynecology clinics at Salmaniya Medical Complex, Manama, Kingdom of Bahrain, between January 2022 and May 2024. The study procedure consisted of two steps. Firstly, blood samples were collected from two groups: gestational diabetic women and normal non-diabetic women. In the second step, insulin levels were measured using a reliable and standardized assay method.

A total of 75 pregnant women between 23 weeks of gestation and term were recruited from both inpatient and outpatient clinics. Among them, 41 pregnant women were diagnosed with gestational diabetes (Test group), while the remaining 34 pregnant women (Control group) had normal blood glucose levels. Prior to participation, all subjects provided their consent. The data collection process involved multiple blood samples.

Initially, RBG levels were measured by collecting random blood samples. Subsequently, the subjects were asked to fast for six to eight hours, after which fasting blood samples were collected to assess FBG and insulin levels. The measurements of blood glucose and insulin were conducted using Atellica Solution Immunoassay and Clinical Chemistry Analyzers (Siemens Healthineers AG, Forchheim, Germany).

The inclusion criteria for participants were pregnant women diagnosed with gestational diabetes based on established diagnostic criteria such as abnormal glucose tolerance test results during pregnancy, and pregnant women with normal non-diabetic status who did not have a history of diabetes or any signs/symptoms suggestive of gestational diabetes. The age range for inclusion was set between 18 and 45 years.

The exclusion criteria for participants were women with pre-existing diabetes, including type 1 or type 2 diabetes diagnosed prior to pregnancy, women with pre-existing medical conditions such as hypertension, renal disease, or autoimmune disorders, as these conditions may independently affect insulin levels, women on medications known to influence insulin levels such as corticosteroids or certain antipsychotic medications. Additionally, women above the age of 45 years were excluded from the study.

By applying these inclusion and exclusion criteria, a cohort of eligible participants was established to investigate the levels of insulin in gestational diabetic women compared to normal non-diabetic women, as well as to assess any variations between Bahraini and non-Bahraini ethnic groups.

Data management and statistical analysis plan

Categorical variables were represented as frequencies and percentages, whereas continuous variables were represented as mean and standard deviation. The Shapiro-Wilk normality test was used to evaluate the distribution of clinical and biological parameters described in this study. Two-sample (independent) t-test was used to test the significant mean differences in the clinical and biological parameters between the tested groups (study group and comparison group). The relationships between parameters were determined using Pearson Correlation Coefficient. The collected data were analyzed using the Statistical Package for Social Sciences (SPSS), version 29 (IBM Corp., Armonk, NY, USA). A p-value < 0.05 was considered statistically significant [18].

## Results

The results showed higher mean values for FBG, RBG, and insulin levels in the test group when compared to the control group. The results of the independent samples t-test indicated that there were statistically significant differences between the two groups in terms of FBG (t=2.265, p=0.026, Cohens d=0.525), RBG (t=2.836, p=0.006, Cohens d=0.628), and insulin (t=2.090, p=0.041, Cohens d=0.567) levels, with medium to large effect size, which illustrated that the readings values of the test group are significantly more for all terms as compared to the control group (Table 1).

**Table 1:** Comparison of FBG, RBG, and insulin levels between the test and the control groups. *Significant, p <0.05.

Factors	Group				Statistics (T value)	p-value	Effect size
	Test (n=41)		Control (n=34)				
	Mean	St. Error	Mean	St. Error			
Fasting blood glucose (FBG) mmol/L	5.40	0.22	4.71	0.20	2.265	0.026*	0.525
Random blood glucose (RBG) mmol/L	6.30	0.33	5.15	0.19	2.836	0.006*	0.658
Insulin μlU/ml	70.43	17.5	30.32	6.7	2.090	0.041*	0.567

When studying the differences between Bahraini and non-Bahraini pregnant women with GDM, the results of the independent samples t-test revealed that there were no statistically significant differences between Bahraini and Non-Bahraini patients in the test group regarding FBG, RBG, and insulin levels (p=0.845, p=0.151, and p=0.072), respectively (Table 2).

**Table 2:** Comparison of FBG, RBG, and Insulin levels in pregnant women with gestational diabetes mellitus (GDM) according to ethnicity of the patients. P-value of Pearson Correlation Coefficient < 0.05 is considered significant.

Factors	Nationality				p-value
	Bahraini (n=12)		Non-Bahraini (n=29)		
	Mean	St. Error	Mean	St. Error	
Fasting blood glucose (FBG) mmol/L	5.33	0.37	5.43	0.28	0.845
Random blood glucose (RBG) mmol/L	5.56	0.37	6.61	0.43	0.151
Insulin μlU/ml	25.23	8.1	97.73	24.24	0.072

There were no statistically significant differences in FBG, RBG, and insulin blood levels (p=0.110, p=0.226, and p=0.133 respectively), between pregnant women with GDM < 30 years old vs those ≥ 30 years old (Table 3).

**Table 3:** Comparison of FBG, RBG, and Insulin levels in pregnant women with gestational diabetes mellitus (GDM) according to age of the patients. P-value of Pearson Correlation Coefficient < 0.05 is considered significant.

Factors	Age group				p-value
	< 30 Years (n=13)		≥ 30 Years (n=28)		
	Mean	St. Error	Mean	St. Error	
Fasting blood glucose (FBG) mmol/L	4.88	0.32	5.65	0.28	0.110
Random blood glucose (RBG) mmol/l	5.71	0.71	6.58	0.35	0.226
Insulin levels μlU/ml	38.87	14.22	96.91	26.03	0.133

## Discussion

This study was an attempt to shed light on the differences in antenatal FBG, RBG, and insulin blood levels between women with and without GDM. Then, we investigated if there were differences in the same variables between Bahraini women with GDM, and comparable non-Bahraini pregnant women with GDM. And finally, we tried to investigate if there were any age-dependent differences in FBG, RBG, and insulin levels in pregnant women with GDM.

From Table 1, our results showed that both FBG and RBG were significantly higher in GDM patients than in non-diabetic pregnant women. Also, patients with GDM showed a significant rise in insulin levels in comparison to non-diabetic pregnant women. Both groups included Bahraini and non-Bahrain women. In the present study, we considered the level of FBG a better indicator for diabetes rather than glycosylated hemoglobin (HbA1c). A previous study by Ghazanfari et al. examined the association between HbA1c and FBG. Their results showed that FBG was a more accurate predictor for GDM than HbA1c, and that FBG was more reliable to separate diabetic from non-diabetic subjects when compared to HbA1c [19].

Our results are in congruence with the previously published data [20-24]. All these studies concluded that the amount of insulin secreted prenatally in GDM patients is a response to the high levels of glucose concentrations. Usman et al. mentioned that even with this high level of insulin during pregnancy, the metabolic abnormalities underlying GDM increased insulin resistance and beta cell defects [25]. Also, results obtained from the study by Homoko et al. supported our findings. Their study revealed that both FBG and insulin levels were significantly higher, and were accompanied by insulin resistance in patients with GDM when compared to non-diabetic control subjects [20]. Several studies attributed systemic insulin resistance during gestation to the rising levels of lactogenic hormones such as prolactin (PRL) and human placental lactogen (hPL) [21-23,26,27].

As for the differences in RBG, FBG, and insulin levels between Bahraini and non-Bahraini pregnant women with GDM, our study revealed that there were no significant differences between these two subgroups. The non-Bahraini groups were from India, Pakistan, Bangladesh, and the Philippines. To our knowledge, the present study is the first study carried out in Bahrain to detect if there are any differences in GDM among different ethnic groups. Nevertheless, several previous studies have shown that there were specific ethnic groups that have a higher incidence of developing GDM [28-31]. These studies demonstrated that Australian Aboriginal, Middle Eastern (Lebanese, Syrian, Iranian, or Iraqi), and Pacific Island women were at a higher risk for developing GDM when compared to women of Western Caucasian origin. Bahraini women are considered a Middle Eastern/Asian group and most of the non-Bahraini women in this study were from South Asia (Indian, Pakistani, and Bengali) and Southeast Asia (Filipino). This could explain, at least in part, why we could not find any difference in the prevalence of GDM in our study. A previous study performed in New York revealed that the prevalence of GDM among South Asian women (Indian, Sri Lankan, Pakistani, Fijian Indian), was generally higher than Southeast Asian (Cambodian, Vietnamese, Thai, Filipino, Malaysian), and East Asian (Chinese, South Korean, Taiwanese and Japanese) women [28-30]. Furthermore, a report by Yuen and Wong mentioned that GDM was higher particularly among women from South Asia and Southeast Asia, compared to Caucasian, African-American and Hispanic communities [32]. Our findings are in congruence with the previous findings on Asian groups as the Kingdom of Bahrain is considered a Middle Eastern Asian country. Our findings are supported by another study by Li et al. which stated that Asian women had a significantly higher risk of developing GDM than European women [33].

Regarding the relationship between age and GDM in pregnant women in the Kingdom of Bahrain, our results showed that although the FBG, RBG, and insulin levels were all higher in pregnant women with GDM aged ≥ 30 years when compared to those with GDM < 30 years, yet these levels were not significantly different between both groups. Our results were not in complete agreement with previously published studies. This could be due to the smaller number of cases in our study compared to larger samples in other studies. Several previous studies showed that there was a relationship between the age of the pregnant woman and GDM [33-36]. In their studies, Li et al. stated that maternal age is a significant risk factor in GDM, and it increases linearly with successive age groups [35]. Additionally, a previous report revealed that obesity and maternal age were the two most important independent factors affecting the risk for development of GDM. The incidence of GDM was high in overweight and in older women [33]. Furthermore, Valadares et al. reported a strong relationship between age and the development of diabetes [36]. Buchanan and Xiang showed that the prevalence of diabetes increases with age, particularly after 45-50 years, and the presence of diabetes also constitutes an important risk factor for cardiovascular disease. Physical inactivity, inadequate diet, and increase in the prevalence of obesity were factors held responsible for the global expansion of diabetes [2]. Gao et al., 2022, investigated the relationship between the age of pregnant women with GDM, the mode of delivery, and the neonatal Apgar score. They concluded that there was a relation between the age of pregnant women with GDM and both the mode of delivery and Apgar score, denoting an increased probability of maternal and fetal adverse outcomes [34].

Limitations and future work

In the present study, the weight of patients was not evaluated and correlated to other findings with GDM. Patients should have been classified according to their weight into normal, overweight, or obese as obesity is a main risk factor in the development of gestational diabetes mellitus. Our future plans include the study of the impact of body weight on pregnant women with GDM in the Kingdom of Bahrain. Additionally, levels of prolactin, human placental lactogen and cortisol levels shall be assessed and correlated to GDM in Bahraini and non-Bahraini women.

## Conclusions

GDM remains a global challenge, with deleterious metabolic and cardiovascular consequences. Insulin resistance is a hallmark of GDM, and proper antenatal care should be provided to minimize the complications of GDM. We showed elevation of blood glucose and insulin levels in pregnant women with GDM. Our results favor the possibility that pregnancy-related circulating factors could be the underlying mechanism in the development of GDM. Age and ethnicity were not related to the development of GDM in the present study.
