# Correction: Insulin Blood Levels in Gestational Diabetes Mellitus in Relation to Ethnicity and Age in the Kingdom of Bahrain: A Case-Control Study

**DOI:** 10.7759/cureus.c204

**Published:** 2024-12-23

**Authors:** Tarik AlShaibani, Wadeea Gherbal, Amer Almarabheh, Diaa Rizk, Elaf Alhakmani, Raghad Alshamrani, Farah AlBahraini, Husain Taha, Amal Hassani, Yahya Naguib

**Affiliations:** 1 Department of Physiology, Arabian Gulf University, Manama, BHR; 2 Department of Obstetrics and Gynecology, Salmaniya Medical Complex, Manama, BHR; 3 Department of Family and Community Medicine, Arabian Gulf University, Manama, BHR; 4 Department of Obstetrics and Gynecology, Arabian Gulf University, Manama, BHR; 5 Department of Internal Medicine, Salmaniya Medical Complex, Manama, BHR; 6 Department of Clinical Physiology, Faculty of Medicine, Menoufia University, Shibin El Kom, EGY

This article has been corrected to change the description of the study in the title and abstract from "cross-sectional" to "case-control". The journal regrets that this error was not identified and corrected prior to publication.

